# Notes and News

**Published:** 1901-04-06

**Authors:** 


### Notes and News.

### HOSPITAL MEETINGS.

Newsvendors' Benevolent and Provident Institution.—The Festival dinner of this institution will be held on Tuesday, June 25th, at Stationers' Hall. The Lord Mayor will preside.

Metropolitan Hospital.—The Anniversary Festival of this hospital will be held on Monday, April 22nd, at the Hotel Metropole, Mr. A. H. Allhusen, M.P., in the chair.

The German Imperial Board of Advisers on Hygiene has held its first meeting. Its functions are to assist the Government on all matters relating to national life, including the housing of the poor, pollution of rivers, food, and numerous other important subjects.

A number of Liverpool residents and shipowners have combined to guarantee the payment of £400 to a professor of tropical medicine and of £105 to a fellow or scholar the posts to be known as the Walter Myers Professorship and Fellowship or Scholarship.

America is always ready with novel and drastic forms of legislation. One of the most recent Bills hails from the Senate of Indiana, which provides for the examination of persons contemplating marriage. The examining board is reported to consist of two physicians, two mothers, and one lawyer.

A new sprudel has been opened at Carlsbad. The spring has been opened out by borings, as the old sprudel became less and less in quantity every year. The steam of the new sprudel is very powerful, rising every few seconds to a height of fifty feet. The yield is 800 litres per minute, but only 300 will be used.

Lieutenant H. E. M. Douglas, of the Royal Army Medical Corps, has been awarded the Victoria Cross for conspicuous bravery. Lieut. Douglas showed great gallantry and devotion in advancing into the open to attend to Captain Gordon, of the Gordon Highlanders, who was wounded, and to Major Robinson and other men (on both occasions he was under severe fire) at Magersfontein in December 1899. Lieut. Douglas has performed many other brave acts.

A magnificent donation has just been anonymously made to the Halifax Infirmary amounting to £7,000. This sum will prove a great boon to the institution, as owing to the great rise in the cost of building the donation of £8,000 generously given by Mr. E. Carlile, J.P., to provide the south wing has proved quite insufficient. Through the recent donation it will be possible to complete and open the wing free of debt.

A new hospital for children has been opened in Paris in the Montmartre district. It contains about 180 cots, some of which are devoted to a crêche, and there are some extra beds for mothers. The cost per bed, including all equipments, was rather more than £280. The hospital, which succeeded the old Hospital Trousseau, is called the Hospital Bretonneau.

In answer to a question by Sir Walter Foster in the House of Commons last Saturday, Mr. Brodrick stated that instructions had been given that all military invalids and soldiers arriving at English ports in ships on which any case of plague had occurred should be isolated and kept under observation for a sufficient period before being allowed to go to their homes or to military camps. We are sorry to note that the outbreak of plague at Cape Town has assumed very considerable proportions.

The new workhouse hospital, to be called the Hospital of St. Luke, built by the Halifax Board of Guardians is about to be opened. The buildings are on a large scale, and are arranged on the pavilion plan for the wards, but these vary in form. There are five pavilions for male and five for female patients. Eight pavilions, of two storeys in height, are oblong, and two are circular and three storeys high. The wards in the oblong pavilions will contain 28 beds in each. There are besides two maternity pavilions. The Nurses' Home is centrally placed, as is also the administration block.

The case of plague which was removed from the hospital ship *Simla* on her arrival at Southampton with invalids from South Africa was apparently another "rat" case. The patient was a baker's mate, and had been in the habit of catching rats, and it is presumed that he caught his disease by handling these creatures. The man is now convalescent. His was the only case on board, and in his case the disease had reached the suppurating stage before his arrival in Southampton, where he was admitted to the South Hants Infirmary suffering from an abscess in the groin requiring operation, the fact of his being affected with plague not having been suspected.

The fine new premises of the Dental Hospital of London in Leicester Square were sufficiently advanced toward completion to be opened last week on the occasion of the annual meeting, though the formal opening will not take place just yet. The erection is a handsome and spacious one, abundantly well lit and admirably adapted to its purpose, experience having enabled provision to be made for all requirements. The building is supplied throughout with electric light and gas, and hot and cold water, and is heated by hot-water pipes. The walls are tiled and the floors of mosaic; there is not a joint or a corner where dust can collect. The cost of the structure is about £50,000, exclusive of the £42,000 paid for the site.

